# A Bootcamp for Transition into Clerkship in a Distributed Campus Model

**DOI:** 10.1007/s40670-026-02678-8

**Published:** 2026-02-26

**Authors:** Michaela Jansen, Ebtesam Islam, Gurvinder Kaur, Sonya Sherrod, Valerie Collins

**Affiliations:** 1 Department of Cell Physiology and Molecular Biophysics, Texas Tech University Health Sciences Center School of Medicine, Lubbock, TX 79430 USA; 2 Department of Medical Education, Texas Tech University Health Sciences Center School of Medicine, Lubbock, TX 79430 USA; 3 Office of Academic Affairs, Texas Tech University Health Sciences Center School of Medicine, Lubbock, TX 79430 USA; 4 Department of Internal Medicine, Texas Tech University Health Sciences Center School of Medicine, Lubbock, TX 79430 USA

**Keywords:** bootcamp, transition to clerkships, introduction to clinical medicine, distributed campus model

## Abstract

**Purpose:**

The transition from preclinical to clinical training represents a critical period in medical education, particularly within multi-campus institutions where students relocate to different clinical sites. Because limited research has examined the effectiveness of transitional programs across distributed medical education sites, this study aimed to evaluate whether a brief, multi-campus transitional bootcamp could improve students’ confidence and preparedness for clerkships. Bootcamps have been used to facilitate transitions in medical education, particularly between phases of the curriculum and between undergraduate and graduate medical education.

**Methods:**

A multi-campus 2-day bootcamp was developed to transition students from the central campus to four clinical training campuses. Campus-specific faculty and senior student preceptors administered different stations on each campus, six stations during the first implementation and 10 during the second year. Participating student surveys were administered immediately before (pre) and after completion (post) of the bootcamp to monitor confidence for each station and overall preparedness for clerkships. Additionally, clerkship director and faculty surveys were administered within the first clerkship period following the bootcamp.

**Results:**

Over the 2-year study period, 191 and 199 students participated, with survey completion exceeding 93% each year. Students consistently reported increased confidence levels across all stations. Following the bootcamp, 99% of students in the first year and 100% in the second year felt more prepared to begin clerkships. Preceptors observed improved readiness and smoother integration into clinical settings during the first clerkship period.

**Conclusions:**

The consistent increase in student confidence and positive preceptor feedback indicated the significant benefit of implementing a focused, 2-day transitional bootcamp. Our data demonstrate that even a brief, structured intervention can improve student confidence, particularly in programs where students transition between campuses. This approach may serve as a practical model for other multi-campus medical schools seeking to support students during this critical period.

**Supplementary Information:**

The online version contains supplementary material available at 10.1007/s40670-026-02678-8.

## Introduction

Phased undergraduate medical education (UME) programs in the United States comprise three distinct stages: Phase 1 of pre-clinical, basic science training, Phase 2 of core-clerkship training, and Phase 3 of advanced clinical training. The transition from pre-clinical to clinical core-clerkship training represents a critical milestone for medical students, marked by increased clinical responsibilities, direct patient care, and a significant shift in learning environment. While this transition is often anticipated with enthusiasm, it is equally recognized as a period of heightened stress and anxiety [1], particularly within multi-campus medical schools where students relocate from a central campus to unfamiliar clinical sites for clerkship training.

To ease this transition, many medical schools have revised their curricula to incorporate active learning strategies [2–4] and have introduced early clinical experiences and skills-based courses during the pre-clerkship phase [5–7]. Although these initiatives have provided some benefits, they have not fully addressed the challenges students face as they enter clerkships [8]. For instance, at our institution, despite the presence of a shared Introduction to Clinical Medicine (ICM) curriculum during the pre-clerkship years at the central campus, student satisfaction with this content has varied by clerkship campus, as reflected in the Association of American Medical Colleges (AAMC) Graduating Student survey (GQ). For some GQ reporting years, intercampus differences in the combined “good” or “excellent” ratings for the item ”How well did the study of the following sciences basic to medicine prepare you for clinical clerkships and electives? Introduction to Clinical Medicine” have approached 20% points. In 2024, for example, overall school satisfaction was 83.8% (compared to 92.4% nationally), with campus-specific satisfaction ranging from 68.8% to 87.2%. The ICM course is a skills-based, longitudinal curriculum that is closely coordinated and integrated with the seven blocks comprising the foundational and organ systems-based pre-clerkship curriculum. It teaches foundational patient communication, history-taking, physical examination, and clinical documentation, while also introducing more advanced communication skills. Additionally, students have reported the desire to revisit foundational clinical concepts from the longitudinal ICM course prior to beginning core-clerkships, especially following the dedicated USMLE Step 1 preparation period. This suggestion was communicated to the authors during mandatory on-campus visits with clerkship students during the bootcamp planning period. Overall, these findings highlight critical gaps in transitional support, gaps that are especially pronounced for students navigating relocation and adjustment to new clinical environments within a multi-campus model.

Various interventions have been proposed to support the transition from pre-clinical to clinical training, including transition-to-clerkship courses [8–15], near-peer mentoring [16, 17], and orientation programs held immediately before the start of clerkships. While these approaches offer clear value, the literature rarely addresses the unique challenges faced by students transitioning to multi-campus clerkship rotations, where unfamiliar personnel (administrators, clerkship directors, faculty) and facilities (simulation centers, training sites) can contribute to increased anxiety, variability in clerkship experiences, and disparities in early clinical performance. Furthermore, many existing interventions span at least a week to achieve meaningful outcomes [8, 16]; however, increasingly condensed curricula, time constraints, and limited institutional resources often make the implementation of week-long or longer transition programs impractical.

In contrast, shorter, 1- to 2-day bootcamps have been shown to be effective in bridging the gap between UME and residencies, by preparing either senior medical students [18, 19] or beginning residents [20, 21]. Despite their success, few programs have systematically applied this model to support the transition between pre-clinical and core-clerkship training, particularly within the unique context of multi-campus schools.

To address these gaps, we implemented a collaborative, focused and standardized bootcamp to facilitate students’ transition to their clinical campuses and increase their confidence as they begin clerkships within a multi-campus medical school model. This bootcamp reinforced essential skills from the pre-clerkship ICM course, including history taking, and physical examination, while also introducing new clerkship-relevant skills such as patient presentations, suturing, image interpretation, and navigation of the campus-specific electronic medical record system (Table 1). The primary goal of this study was to assess the effectiveness of the bootcamp in improving learner confidence levels and facilitating successful integration into the first core-clerkship period across multiple campuses. Additionally, this initiative was intentionally designed to actively engage stakeholders from all clinical sites, promote consistent preparation among learners, and serve as a potential model for enhancing transitional support within multi-campus medical school settings.

**Table 1:** Student confidence ratings pre- and post-bootcamp by station for 2024. Responses were collected on a 1–5 Likert scale and are summarized as median (IQR), where IQR represents the 25th–75th percentiles (Q1–Q3). Δ median indicates the post minus pre difference in medians. Because pre- and post-responses were not linkable at the individual level, groups were compared using two-sided Wilcoxon rank-sum (Mann–Whitney) tests. Effect size is reported as Cliff’s delta

	Station	*n* (Pre)	*n* (Post)	Pre, Median (IQR)	Post, Median (IQR)	Δ median	Mann–Whitney U	*p* (raw)	Cliff’s δ
1	Knot tying skills (EPA 12, Perform general procedures of a physician).	185	183	2 (1–2)	4 (3–4)	2	5,086	< 0.0001	0.700
2	Ultrasound image acquisition (EPA 12, Perform general procedures of a physician).	185	183	2 (2–3)	4 (3–4)	2	7,211	< 0.0001	0.574
3	Active participation in rounding (EPA 5, Document a clinical encounter; EPA 6, Provide an oral presentation of a clinical encounter).	185	183	3 (2–3)	4 (3–4)	1	7,563	< 0.0001	0.553
4	Clinical reasoning (EPA 2, Prioritizing a differential diagnosis following a clinical encounter).	185	183	3 (2–3)	4 (3–4)	1	8,261	< 0.0001	0.512
5	Relationship centered communication skills (EPA 1, Gather a history and perform a physical exam).	185	183	4 (3–4)	4 (4–5)	0	10,075	< 0.0001	0.405
6	Handling communication barriers (EPA 1, Gather a history and perform a physical exam).	185	183	3 (3–4)	4 (4–5)	1	7,328	< 0.0001	0.567

## Methods

### Ethics

This study was approved by the Texas Tech University Health Sciences Center-Quality Improvement Review Board under protocol number: QI-24,024.

### Bootcamp Development

For the first iteration, a six-station bootcamp was developed at the Texas Tech University Health Sciences Center School of Medicine (TTUHSC SOM) in Lubbock, Texas. This 2-day event was scheduled immediately following the campus orientation to serve as a targeted transition to the core clerkships. The bootcamp was designed with explicit alignment to the AAMC Entrustable Professional Activities (EPAs) to ensure that the objectives directly address core competencies expected of students entering clinical training (Table 1). The development process was highly collaborative. Following presentation of the proposal to both the Continuous Quality Improvement Committee and the Curriculum Renewal Steering Committee, the Associate Dean for Medical Education and Accreditation partnered with the Associate Dean for Clinical Education and the Coordinator for Special Projects to draft the initial framework for the bootcamp. Next, student perspectives were solicited during class meetings held at each campus. Subsequently, through a series of video-conference and in-person meetings involving the ICM course director, clerkship directors and coordinators from all four campuses, simulation center representatives from all campuses, and administrators from all campuses, bootcamp objectives, including those specific to each of the six stations, were developed through a collaborative process and formally approved by the School of Medicine curriculum committee.

Similarly, the detailed planning for each station, including setting, resources and staffing (e.g., near peers, residents, faculty), was carried out through collaborative video-conference meetings. The process was aimed towards having a standardized and consistent experience across all four simulation centers and campuses.

### Bootcamp Objectives

The following are the objectives for the bootcamp in 2024 and 2025:

1. Demonstrate Proficiency in Patient Interaction and Communication.
2. Develop Sound Diagnostic Skills and Formulate Differential Diagnoses.
3. Implement Basic Clinical Procedures with Competence and Confidence.
4. Apply Ethical Decision-Making and Professionalism in Clinical Practice.
5. Recognize and Respond to Medical Emergencies with Competence.
6. Exhibit a Patient-Centered Approach to Healthcare.
7. Demonstrate Proficiency in Medical Documentation and Record-Keeping.

### Bootcamp Implementation

The bootcamp was implemented twice for two consecutive years during orientation week, immediately preceding the start of core clerkships. In both 2024 and 2025, the bootcamp was delivered over 2 days for the Class of 2026 and the Class of 2027, respectively. Based on student and facilitator feedback from the 2024 iteration, the 2025 bootcamp was expanded to enhance the overall experience and better address student needs. Specifically, the number of stations increased from six to 10, reflecting both refinement of existing content and the addition of new topics. Stations in 2024 were: Knot tying skills and Ultrasound image acquisition, Active participation in rounding, Clinical reasoning, Relationship centered communication skills, Handling communication barriers, and Physical examination practice. In 2025, these stations were retained in principle but revised and expanded to 10 stations, as follows: Scrubbing and suturing, Ultrasound, Image interpretation, Relationship centered communication, Physical exam practice, Developing differential diagnosis, Electronic medical records, Rounding and presenting patients, Barriers to care, and Tips and tricks. To foster peer connection and simulate clinical team structures, students were grouped based on the clerkships they were entering together.

### Data Collection

To assess the educational impact of each bootcamp iteration, students were asked to complete both a pre- and post-survey instrument. The pre-survey was administered immediately before the bootcamp began, and the post-survey was completed at bootcamp conclusion. For each station, students reported their confidence levels both before and after the bootcamp, allowing for measurement of self-perceived skill development. For each station, students were asked to “Rate your confidence in [station title including EPAs]” using a Likert scale ranging from 1 (*lowest confidence*) to 5 (*highest confidence*). Additionally, the post-survey included a global question that prompted students to reflect on whether they felt better prepared for their upcoming clerkships as a result of the bootcamp experience (“After the bootcamp, I feel more prepared for clerkships. Answer choices: Disagree, Agree”).

Additionally, clerkship directors, faculty, and residents were surveyed to evaluate the perceived real-world impact of the bootcamp on student preparedness during the first core-clerkship period (“Overall, students were better prepared to start their first clerkship. Answer choices: Better than previous years’ students, Equal to previous years’ students, worse than previous years’ students, N/A”). The instructor survey included an optional open-text item for general comments, feedback and suggestions. Comments were reviewed and summarized descriptively to identify recurring themes and improvement suggestions (no formal qualitative coding framework was applied). This survey opened during the last week of the first clerkship rotation and remained available for two additional months to capture broad faculty and resident perspectives on student performance and readiness. Response rates for the 2024 clinical instructor survey cannot be calculated because the survey was distributed by clerkship coordinators without central tracking of recipients. In 2025, the survey was centrally distributed to 891 clinical instructors.

### Statistical Analysis

All survey responses were collected anonymously to promote unbiased feedback. Raw survey response data were exported from Qualtrics, a secure web-based survey platform. Descriptive statistics and data analysis were performed using GraphPad Prism 10 (GraphPad Prism version 10.0.0 for Windows, GraphPad Software, Boston, Massachusetts USA, www.graphpad.com). Cliff-s delta was calculated using (δ = count[post > pre] − count[post < pre])/n_pre_n_post_.

### Data Visualization

Confidence ratings were collected on a 5-point Likert scale (1 = *lowest confidence*, 5 = *highest confidence*) pre- and post-bootcamp. For descriptive visualization, response distributions were converted to within-station percentages and displayed as diverging stacked bar charts, grouping 1–2 as lower confidence (negative direction) and 3–5 as neutral-to-higher confidence (positive direction).

## Results

**Fig. 1:**
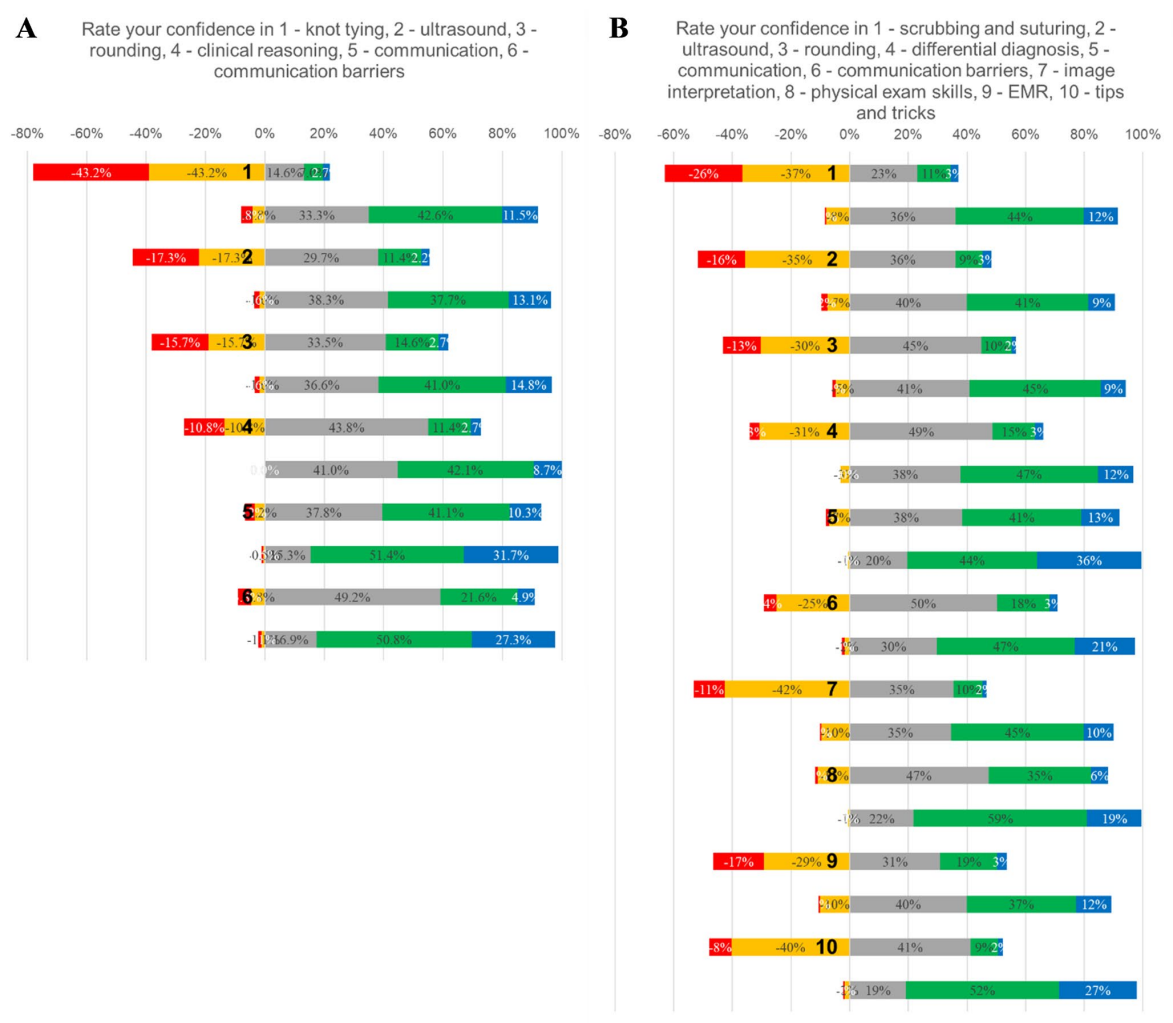
Pre- and Post-Bootcamp Confidence Levels for each Station with the numbering of stations as in Tables 1 and 2. (**A**) 2024 (**B**) 2025. Diverging stacked bar chart of student confidence ratings (Likert 1–5) for Stations 1–6 (A) or Stations 1-10 (B) before/pre and after/post the bootcamp. For each station, the proportion of responses in Categories 1–2 is displayed to the left of zero (negative direction), and Categories 3–5 are displayed to the right of zero (positive direction). Bars are horizontal and ordered as Station 1 Pre, Station 1 Post, Station 2 Pre, Station 2 Post, continuing through all stations. Values represent the percentage of responses in each Likert category; each bar sums to 100%. Likert scale rating colors indicated as 1-red, 2-orange, 3-grey, 4-green, and 5-blue

The total number of participants in the bootcamp was 191 in 2024 and 199 in 2025, respectively. The pre-survey completion rates were 97% and 93%, and the post survey completion rates were 96% and 94% for 2024 and 2025, respectively. The bootcamp was effective in increasing the students’ confidence levels to start clerkships (Figs. 1). The percentage of students indicating they felt more confident to start clerkships after the bootcamp was 99% and 100% in 2024 and 2025, respectively.

Tables 1 and 2 summarize student pre- and post-bootcamp ratings (Likert 1–5) by station, reported as median (IQR) and compared using two-sided unpaired Wilcoxon rank-sum (Mann–Whitney) tests (pre/post responses were not linkable). In the first implementation in 2024 (Tables 1; 6 stations), post-bootcamp ratings increased across all stations, with median gains ranging from 0 to 2 points; the largest shifts occurred in stations with lower baseline ratings (e.g., median increasing from 2 to 4). All station comparisons were statistically significant (*p*-values < 0.0001), with moderate-to-large effect sizes (Cliff’s δ = 0.41–0.70). In the second year 2025 (Tables 2; 10 stations), a similar pattern was observed across the expanded station set, with median gains of 0 to 2 points and statistically significant pre/post differences across all stations (*p-*values < 0.0001). Effect sizes for the six stations shared across both years were highly consistent: δ ranged from 0.41 to 0.70 in Year 1 and 0.36–0.64 in Year 2, with year-to-year differences per station small (approximately − 0.06 to + 0.03). Across both years, the station related to communication (Station 5), which is an area of significant focus during ICM, showed the smallest effect (Year 1 δ = 0.41; Year 2 δ = 0.36). The largest effect was observed in the scrubbing/suturing station (Station 1) in Year 1 (δ = 0.70) and in the tips and tricks for clerkship success station (Station 10) in Year 2 (δ = 0.78); among the stations shared across both years, Station 1 remained the largest effect in Year 2 (δ = 0.64).Newly added stations in Year 2 also showed moderate-to-large effects (δ = 0.42–0.78), including the largest effect in Station 10 (δ = 0.78), supporting reproducible improvements in student-perceived readiness across consecutive cohorts.

**Table 2:** Student Confidence Ratings Pre- and Post-Bootcamp by Station for 2025. Responses were collected on a 1–5 Likert scale and are summarized as median (IQR), where IQR represents the 25th–75th percentiles (Q1–Q3). Δ median indicates the post minus pre difference in medians. Because pre- and post-responses were not linkable at the individual level, groups were compared using two-sided Wilcoxon rank-sum (Mann–Whitney) tests. Effect size is reported as Cliff’s delta. Effect size difference for comparable Stations 1–6 between 2024 and 2025 is reported as Δ Cliff’s delta

	Station	*n* (Pre)	*n* (Post)	Pre, Median (IQR)	Post, Median (IQR)	Δ median	Mann–Whitney U	*p* (raw)	Cliff’s δ	Δ Cliff’s δ 2025 − 2024
1	Knot tying skills (EPA 12, Perform general procedures of a physician).	186	188	2 (1–3)	4 (3–4)	2	6,263	< 0.0001	0.642	-0.058
2	Ultrasound image acquisition (EPA 12, Perform general procedures of a physician).	186	188	2 (2–3)	4 (3–4)	2	7,826	< 0.0001	0.552	-0.022
3	Active participation in rounding (EPA 5, Document a clinical encounter; EPA 6, Provide an oral presentation of a clinical encounter).	185	188	3 (2–3)	4 (3–4)	1	7,507	< 0.0001	0.568	0.015
4	Clinical reasoning (EPA 2, Prioritizing a differential diagnosis following a clinical encounter).	185	188	3 (2–3)	4 (3–4)	1	8,056	< 0.0001	0.537	0.025
5	Relationship centered communication skills (EPA 1, Gather a history and perform a physical exam).	185	188	4 (3–4)	4 (4–5)	0	11,062	< 0.0001	0.364	-0.041
6	Handling communication barriers (EPA 1, Gather a history and perform a physical exam).	185	188	3 (2–3)	4 (3–4)	1	7,546	< 0.0001	0.566	-0.001
7	.Image interpretation (EPA 3, Recommend and interpret common diagnostic and screening tests).	186	188	2 (2–3)	4 (3–4)	2	7,012	< 0.0001	0.599	
8	Physical exam skills (EPA 1: Gather a history and perform a physical exam).	186	188	3 (3–4)	4 (4–4)	1	10,115	< 0.0001	0.421	
9	Using electronic medical records (EPA 5, Document a clinical encounter in the patient record).	185	188	3 (2–3)	3 (3–4)	0	9,600	< 0.0001	0.448	
10	Tips and tricks for clerkship success.	184	188	3 (2–3)	4 (4–5)	1	3,866	< 0.0001	0.776	

In 2024, the clinical instructor survey yielded 45 responses, including responses from all core-clerkship campuses and all specialties. However, due to the small number of respondents, not all clerkship specialties were represented at each campus. Of the respondents, 24% were from the Amarillo campus, 9% from the Covenant campus, 48% from the Lubbock campus, and 29% from the Odessa campus. Almost half of the respondents (44%) were clerkship directors, 38% clerkship faculty, and 18% residents. Less than half (40%) reported being directly involved in the administration of the bootcamp on their respective campuses. When asked to compare student preparedness to previous years, 78% of respondents from the Amarillo campus, 100% for the Covenant campus, 47% for the Lubbock campus, and 60% for the Odessa campus indicated that students were better prepared, and no respondents indicated that students were less prepared than in previous years. Equal preparation was indicated by 22% of Amarillo campus, 53% of Lubbock campus, and 40% of Odessa campus respondents.

Similarly, in 2025, the clinical instructor survey was conducted at the end of the first clerkship period. The survey was sent to 891 clinical instructors and yielded 102 responses, an 11% response rate, covering 22 of the 23 core clerkship sites. Of the respondents, 15% were from the Amarillo campus, 16% from the Covenant campus, 48% from the Lubbock campus, and 22% from the Odessa campus. About one-fifth (18%) of respondents were clerkship directors, 56% clerkship faculty, and 27% residents. Thirty percent reported being directly involved in the administration of the bootcamp on their respective campuses. When asked to compare student preparedness to that in previous years, 42% of respondents from the Amarillo campus, 43% for the Covenant campus, 55% for the Lubbock campus, and 26% for the Odessa campus indicated that students were better prepared. Equal preparation was indicated by 42% of Amarillo campus, 26% of Lubbock campus, and 58% of Odessa campus respondents.

Faculty and clinical observers consistently reported that bootcamp participants demonstrated increased confidence, preparedness, and adaptability in clinical environments, including rounds, clinic, and the operating room. We observed substantial variation across campuses in the proportion of clinical instructors who reported improved student preparedness after implementation of the bootcamp. This distribution mirrored previously observed campus-level differences in student satisfaction with the pre-clerkship ICM curriculum, which we hypothesized could have been driven in part by the limited involvement of clinical instructors at geographically distant sites. By engaging campus-based faculty and staff to deliver the bootcamp, the intervention helped align expectations across sites and appeared to attenuate intercampus differences. In narrative comments, instructors noted that students were more comfortable engaging in patient interactions and formulating clinical differentials, with several evaluators noting that their performance was comparable to that of more experienced third-year students and some noting it was not immediately apparent that this was their first clinical rotation. Students also demonstrated improved receptiveness to feedback, adaptability, and patient-centered behaviors. While some evaluators highlighted ongoing challenges, such as difficulties with documentation, history-taking, and prioritizing diagnoses, the overall impression was that the bootcamp significantly improved students’ readiness to integrate into their clerkship environments. Suggestions for program improvement included distributing the content over additional days to reduce fatigue and support retention.

## Discussion

The transition from pre-clinical to clinical training remains a recognized challenge, particularly in multi-campus medical schools where students must adapt not only to new clinical responsibilities but also to unfamiliar environments. Clerkship directors have frequently reported that incoming third-year students lack readiness in core areas such as communication, physical examination, clinical reasoning, professionalism, and understanding of life cycle stages [22]. Students themselves echo these concerns, reporting feeling underprepared, which undermines their confidence and early performance [23]. Similar findings have been reported internationally. A survey of fourth-year students at Maastricht Medical School highlighted persistent challenges, including uncertainty about expectations, difficulty applying theoretical knowledge to patient care, increased workload, and limited study time. While students reported moderate preparedness in knowledge and physical examination skills, many lacked the practical experience necessary for clinical settings [24]. These consistent gaps highlight the need for structured, targeted interventions to bridge classroom learning and clinical practice during this critical transition.

To address these challenges, many medical schools have implemented transition courses, orientations or preparatory sessions immediately prior to clerkships. These initiatives aim to reinforce clinically relevant knowledge, provide opportunities to practice key skills with feedback, and offer structured support to ease the transition into clinical environments. Two national surveys illustrate the evolution of such efforts. In 2003, a survey of 56 medical schools (56% response rate) identified 30 transition courses, most (83%) lasting 1 week or less. These courses emphasized introducing new clinical skills, reinforcing preclinical knowledge, and promoting student well-being, with topics such as technical skills, safety protocols, orientation to clinical settings, and stress management frequently addressed. Over half these courses (12) facilitated peer interaction with students who had completed clerkships. By 2010, 88% of responding U.S. (78/126) and Canadian (5/16) medical schools reported having implemented transition courses [16]. While participation was often mandatory, only 35% of programs awarded grades, and 41% formally evaluated student performance. The core objectives consistently emphasized clinical skill development, application of preclinical knowledge, and student well-being. Course content also included clinical tasks (41%), workplace culture (37%), and interpersonal skills (17%). Didactic sessions were nearly universal (98%), and 74% incorporated hands-on practice, though only 21% included experiences in clinical settings. Senior medical students participated in 82% of these courses, and over half engaged multiple clerkship departments [8]. Despite their widespread use, many transition courses required at least 1 week and often longer to achieve meaningful outcomes [9, 10, 11, 12, 25, 26, 27]. Such time frames are increasingly impractical given condensed curricula, time constraints, and limited resources. In response to these constraints, most recent literature has shifted towards shorter, specialty-specific bootcamps. For example, otolaryngology bootcamps for first- and second-year medical students have employed a brief, 3-hour format that combined didactics with supervised skills practice [28]. Similarly, neurosurgical and OBGYN bootcamps have focused on preparing learners for specialty-specific sub-internships through 1-day interventions that included lectures and cadaver-based or procedural skills training [29, 30]. While effective, these programs targeted narrow learner populations and specialty-defined outcomes rather than global readiness for core clerkships. While bootcamps have been widely described as a strategy to bridge the transition from UME to residency, with positive outcomes in areas such as technical skill acquisition and confidence building [18], few programs have systematically applied this model to support the transition from pre-clinical and core-clerkship training, particularly within multi-campus institutions.

In this study, we describe the successful implementation of a standardized, collaborative bootcamp to ease the transition to core clerkships within a multi-campus medical school. Our findings help address an under-studied aspect of clerkship transitions in multi-campus programs, where site-to-site differences in personnel and clinical environments may affect early learner experience and performance. The bootcamp reinforced essential pre-clerkship skills, such as history taking and physical examination, while introducing new, clerkship-relevant competencies, including patient presentations, suturing, image interpretation, and navigation of campus-specific electronic medical records. O’Brien et al. proposed that grouping students by their first clerkship site may enhance practical skills training [8]. Following this framework, our bootcamp divided students into groups paired by their initial clerkship site. O’Brien et al. also reported that learning from senior medical students represented the most effective element of transition courses (40% of 60 courses with responses) [8]. Similarly, Ramakrishnan et al. implemented a near-peer (sideways) mentorship model during a 1-hour internal medicine clerkship orientation bootcamp [31]. Using pre- and post- bootcamp surveys, the authors demonstrated a significant increase in student confidence across all 10-core internal medicine clerkship domains, supporting the effectiveness of a structured student-led bootcamp in improving clerkship preparation. At our institution, senior medical students facilitated skills stations alongside faculty and provided informal mentorship on adjusting to clerkships. Our findings demonstrate that the bootcamp successfully achieved its primary objective of increasing student confidence and perceived readiness for core-clerkships. The conclusion was supported not only by student self-assessments but also by faculty and resident observations, which indicated noticeable improvements in students’ clinical performance and readiness. Together, these findings highlight the bootcamp’s effectiveness in preparing students for the demands of Phase 2 clinical education.

A common limitation of the transition courses is the reliance on variable assessments, such as attendance or self-perception surveys, rather than objective measures of clinical competence. To truly support workplace readiness, evaluation should focus on students’ ability to engage in key clinical activities early in clerkships. A recently published study addressing this gap introduced a 2-week transition course and demonstrated measurable improvements in student performance [28]. Students who participated in the transition course and simulation showed statistically significant improvements in summative performance across clerkships compared to that of the standard group, with the most pronounced impact observed during the early clerkships of the academic year. Our findings align with these results and further demonstrate the value of structured transition initiatives. Instructors’ narrative feedback complemented student self-assessments and suggested improved early clerkship readiness following the bootcamp, while also identifying targeted areas for iterative improvement. There was strong consensus in support of making the bootcamp a recurring component of the curriculum.

Several limitations should be acknowledged. The instructor survey in 2025 had a response rate of 11% and was likely also low in 2024, even though we do not know the number of recipients for that year due to its decentralized distribution. Interpretation of the 2025 instructor survey item comparing student performance during the first clerkship period with prior years is limited by an ambiguous reference group. Because the comparison necessarily included students who had participated in the 2024 bootcamp, it is unclear whether instructors anchored their responses to cohorts from the pre–bootcamp era or to students trained under the 2024 iteration rather than the 2025 model. Concurrent updates to the longitudinal ICM curriculum for the classes of 2025 and 2026 may have contributed to students’ overall preparedness, making it difficult to isolate the specific impact of the bootcamp. However, the time gap between the end of the ICM course and the commencement of Phase 2, contributed to students forgetting the material, according to students during the authors’ on-campus visits with clerkship students. The low response rate for the post-first-clerkship faculty survey during the first implementation year may have introduced response bias, limiting generalizability of those findings. Additionally, variations in resources and logistics across the four participating sites posed challenges to ensuring uniform delivery of the bootcamp experience, which may have affected outcomes.

Despite these limitations, implementing the bootcamp across multiple campuses underscored the value of collaborative efforts in distributed medical education. Shared development of curriculum resources such as standardized case studies and simulation exercises helped ensure a consistent educational experience, irrespective of site-specific constraints. Moreover, the process fostered stronger inter-campus faculty relationships and promoted a more unified approach to clinical skills training. These collaborative practices offer a promising model for other distributed medical education programs seeking to enhance student preparation while leveraging collective institutional strengths. Future efforts should focus on improving data collection strategies, particularly for post-clerkship faculty evaluations, to ensure more comprehensive and representative outcome assessments. Longitudinal studies are also needed to assess the sustained impact of the bootcamp on students’ clinical performance and confidence as they progress through medical training. These future directions will be essential for refining the bootcamp model and ensuring its continued relevance and effectiveness in supporting student success.

## Conclusion

In summary, our findings support a brief, standardized, multi-campus bootcamp as a feasible and effective approach to bridging the transition into core clerkships within distributed medical education. By pairing students by initial clerkship site and leveraging campus-based faculty and near-peer preceptors, the bootcamp program reinforced foundational clinical skills, introduced clerkship-relevant tasks, and aligned expectations across geographically separated sites. Consistent gains in student confidence and preparedness that were also corroborated by faculty and resident observations suggest that even a 2-day intervention can meaningfully improve early clerkship readiness while offering a practical, scalable model for multi-campus institutions facing tight curricular time and resource constraints. Given these results, the bootcamp will remain an annual component of the curriculum, with ongoing, data-informed refinements and targeted iterative enhancements implemented each year to optimize content, delivery, and learner experience.

## Supplementary Information

Below is the link to the electronic supplementary material.

Supplementary Material 1 (DOCX 1.05 MB)
