# Supplementary material for: A Bootcamp for Transition into Clerkship in a Distributed Campus Model
Source: Med Sci Educ. 2026 Feb 26;36(2):963–72. doi: 10.1007/s40670-026-02678-8 (PMC13197541; doi:10.1007/s40670-026-02678-8)
Supplement: Supplementary file 1 — Supplementary Material 1 (DOCX 1.05 MB) [file 40670_2026_2678_MOESM1_ESM.docx]

# Appendix

Appendix 1: Detailed Confidence Ratings by Campus for 2024: Pre- and Post-Bootcamp Confidence Levels for each Station. Diverging stacked bar chart of student confidence ratings (Likert 1–5) for Stations 1–6 before/pre and after/post the boot camp. For each station, the proportion of responses in categories 1–2 is displayed to the left of zero (negative direction), and categories 3–5 are displayed to the right of zero (positive direction). Values represent the percentage of responses in each Likert category; each bar sums to 100%. Likert Scale rating colors indicated as 1-red, 2-orange, 3-grey, 4-green, and 5-blue. The top five rows depict the pre-test results for the campuses Ama, Cov, Lbb HSC, Ode, and all campuses combined (“Total Pre-Test”). And the bottom five depict the post test results similarly.

Appendix 2: Detailed Confidence Ratings by Campus for 2025:

Appendix 3: Individual Station Objectives 2024

- Knot tying & suturing skills
  - - Identify and select appropriate types of knots for different surgical scenarios.
    - Demonstrate proper hand positioning and technique for tying secure and reliable knots.
    - Apply different knot-tying methods, including square knots and surgeon’s knots.
    - Understand the principles of aseptic technique and maintain a sterile field during suturing.
    - Foster a growth mindset by recognizing that proficiency in surgical skills is an ongoing process.
- Ultrasound Skills
  - - Demonstrate proper handling and manipulation of ultrasound probes.
    - Practice adjusting depth, gain, and focus settings for optimal image quality.
    - Develop proficiency in obtaining standard views for different organs and regions.
    - Apply ultrasound skills to support clinical decision-making in different medical specialties
    - Utilize feedback to identify areas of improvement and develop strategies for ongoing learning.
- X-ray Skills
  - - Identify and differentiate key anatomical structures in X-ray images, including bones, organs, and soft tissues.
    - Practice recognizing normal variations in anatomy to enhance proficiency in identifying abnormalities or pathologies.
- Relationship Centered Communication
  - - Cultivate empathetic and compassionate communication skills to establish a genuine connection with patients.
    - Explore techniques for understanding and validating the emotional experiences of patients, fostering a supportive and trusting healthcare environment.
    - Improve active listening skills to ensure a thorough understanding of patients' concerns, values, and preferences.
    - Acquire strategies for navigating difficult or sensitive conversations with patients and their families.
- Physical Exam Practice
  - Cardiac exam
    - Identify and locate anatomical landmarks for cardiac examination.
    - Demonstrate proficiency in auscultation of heart sounds, identifying normal and abnormal findings.
    - Assess peripheral pulses and recognize signs of cardiac abnormalities during examination.
  - Lung exam
    - Perform a systematic lung examination, including inspection, palpation, and auscultation.
    - Recognize normal breath sounds and identify abnormal findings such as crackles, wheezes, and decreased breath sounds.
    - Evaluate respiratory effort and chest expansion during the examination.
  - Abdominal exam
    - Demonstrate proper techniques for inspecting, auscultating, percussing, and palpating the abdomen.
    - Identify normal and abnormal abdominal sounds and recognize signs of organomegaly or tenderness.
    - Assess for signs of hernias, masses, or other abdominal abnormalities.
  - Neurological exam
    - Perform a comprehensive neurological examination, including cranial nerve assessment, motor and sensory function, and reflex testing.
    - Recognize signs of abnormal neurological findings and relate them to potential underlying conditions.
    - Demonstrate proficiency in assessing gait, coordination, and mental status
  - Musculoskeletal exam
    - Conduct a systematic musculoskeletal examination, including inspection, palpation, and range of motion assessment.
    - Identify common musculoskeletal abnormalities such as joint swelling, deformities, and muscle weakness.
    - Evaluate functional status and assess the impact of musculoskeletal conditions on daily activities.
  - HEENT exam
    - Demonstrate proficiency in conducting a systematic examination of the head, eyes, ears, nose, and throat.
    - Identify normal and abnormal findings in the HEENT system, including lymph nodes facial structures, and sensory organs.
  - Pelvic Exam
  - Identify and locate anatomical landmarks for pelvic speculum examination, including the introitus, vaginal walls, cervix, and fornices.

ii. Demonstrate proficiency in the insertion and manipulation of the speculum, ensuring patient comfort and proper visualization of the cervix.

iii. Assess the cervix for normal characteristics such as color, position, size, and presence of discharge or lesions, while also identifying abnormal findings

- - Breast Exam
  - Identify and locate anatomical landmarks for breast examination

ii. Demonstrate proficiency in palpation techniques for assessing breast tissue

iii. Assess breast tissue for characteristics such as texture, size, shape, symmetry, and presence of lumps, nodules, or skin changes, while also recognizing signs of abnormalities such as masses or nipple discharge.

- Clinical Reasoning
  - - Explore the cognitive processes involved in clinical reasoning, including pattern recognition, hypothesis generation, and problem-solving.
    - Demonstrate the ability to systematically approach patient cases, considering relevant history, physical examination findings, and diagnostic data.
    - Practice organizing and synthesizing clinical information to formulate a comprehensive patient assessment.
    - Identify and apply key diagnostic principles, including recognizing red flags, considering differential diagnosis, and utilizing diagnostic tests effectively.
    - Demonstrate effective decision-making in situations with incomplete or ambiguous information.
- EMR/Rounding
  - Electronic Medical Records
    - Navigate through various sections of the EMR system efficiently.
    - Utilize EMR functionalities for tasks such as reviewing patient history, orders, results, and progress notes.
    - Demonstrate an understanding of HIPAA regulations and the importance of patient confidentiality.
    - Utilize search functions and filters to find specific clinical data efficiently.
  - Rounding/Presenting patients
    - Develop and deliver concise and structured patient presentations during rounds.
    - Include pertinent information such as chief complaint, history, physical examination findings, assessment, and plan.
    - Practice adjusting the level of detail in presentations for different clinical scenarios.
    - Demonstrate efficient time management skills when presenting patients on rounds.
  - Overall
    - Engage in simulated patient rounds to apply EMR skills and patient presentation techniques in a realistic setting.
    - Engage in reflective practice to analyze the impact of accurate and thorough documentation on patient care.
    - Identify strategies to balance thorough documentation with the demands of a busy clinical setting.
- Barriers to Care
  - Objectives
    - Recognize the impact of building rapport on patient trust, satisfaction, and overall healthcare outcomes.
    - Identify common communication barriers in healthcare, including language differences, cultural nuances, cognitive impairments, and sensory deficits.
    - Develop awareness of personal biases and potential barriers to effective communication.
    - Practice using body language, facial expressions, and gestures to convey empathy and understanding.
    - Explore the use of technology, such as translation services and communication aids, to overcome language and hearing barriers.
    - Emphasize the importance of involving patients in shared decision-making.
    - Practice collaborative communication to empower patients to actively participate in their healthcare.
    - Participate in simulated patient interactions to apply communication skills in realistic scenarios.
    - Engage in reflective practice to analyze personal communication strengths and areas for improvement.

Appendix 4: Individual Station Objectives 2025

1. Scrubbing & Suturing (80 minutes)
   1. Understand the principles of aseptic techniques and the importance of maintaining sterility.
   2. Review the proper procedure for scrubbing, gowning, and gloving to prevent contamination.
   3. Identify and select appropriate types of knots for different surgical scenarios.
   4. Demonstrate proper hand positioning and technique for tying secure and reliable knots.
   5. Apply different knot-tying methods, including square knots and surgeon’s knots.
   6. Foster a growth mindset by recognizing that proficiency in surgical skills is an ongoing process.
2. Ultrasound (80 minutes)
   1. Demonstrate proper handling and manipulation of ultrasound probes.
   2. Practice adjusting depth, gain, and focus settings for optimal image quality.
   3. Develop proficiency in obtaining standard views for different organs and regions.
   4. Apply ultrasound skills to support clinical decision-making in different medical specialties
   5. Utilize feedback to identify areas of improvement and develop strategies for ongoing learning.
3. Image Interpretation (60 minutes)
   1. Identify and differentiate key anatomical structures in X-ray images, including bones, organs, and soft tissues.
   2. Practice recognizing normal variations in anatomy to enhance proficiency in identifying abnormalities or pathologies.
4. Relationship Centered Communication (115 minutes)
   1. Cultivate empathetic and compassionate communication skills to establish a genuine connection with patients.
   2. Explore techniques for understanding and validating the emotional experiences of patients, fostering a supportive and trusting healthcare environment.
   3. Improve active listening skills to ensure a thorough understanding of patients' concerns, values, and preferences.
   4. Acquire strategies for navigating difficult or sensitive conversations with patients and their families.
5. Physical Exam Practice (115 minutes)
   1. Cardiac exam
      1. Identify and locate anatomical landmarks for cardiac examination.
      2. Demonstrate proficiency in auscultation of heart sounds, identifying normal and abnormal findings.
      3. Assess peripheral pulses and recognize signs of cardiac abnormalities during examination.
   2. Lung exam
      1. Perform a systematic lung examination, including inspection, palpation, and auscultation.
      2. Recognize normal breath sounds and identify abnormal findings such as crackles, wheezes, and decreased breath sounds.
      3. Evaluate respiratory effort and chest expansion during the examination.
   3. Abdominal exam
      1. Demonstrate proper techniques for inspecting, auscultating, percussing, and palpating the abdomen.
      2. Identify normal and abnormal abdominal sounds and recognize signs of organomegaly or tenderness.
      3. Assess for signs of hernias, masses, or other abdominal abnormalities.
   4. Neurological exam
      1. Perform a comprehensive neurological examination, including cranial nerve assessment, motor and sensory function, and reflex testing.
      2. Recognize signs of abnormal neurological findings and relate them to potential underlying conditions.
      3. Demonstrate proficiency in assessing gait, coordination, and mental status
   5. Musculoskeletal exam
      1. Conduct a systematic musculoskeletal examination, including inspection, palpation, and range of motion assessment.
      2. Identify common musculoskeletal abnormalities such as joint swelling, deformities, and muscle weakness.
      3. Evaluate functional status and assess the impact of musculoskeletal conditions on daily activities.
   6. HEENT exam
      1. Demonstrate proficiency in conducting a systematic examination of the head, eyes, ears, nose, and throat.
      2. Identify normal and abnormal findings in the HEENT system, including lymph nodes facial structures, and sensory organs.
   7. Pelvic Exam
      1. Identify and locate anatomical landmarks for pelvic speculum examination, including the introitus, vaginal walls, cervix, and fornixes.
      2. Demonstrate proficiency in the insertion and manipulation of the speculum, ensuring patient comfort and proper visualization of the cervix.
      3. Assess the cervix for normal characteristics such as color, position, size, and presence of discharge or lesions, while also identifying abnormal findings
   8. Breast Exam
      1. Identify and locate anatomical landmarks for breast examination
      2. Demonstrate proficiency in palpation techniques for assessing breast tissue
      3. Assess breast tissue for characteristics such as texture, size, shape, symmetry, and presence of lumps, nodules, or skin changes, while also recognizing signs of abnormalities such as masses or nipple discharge.
6. Developing Differential Diagnosis (115 minutes)
   1. Explore the cognitive processes involved in clinical reasoning, including pattern recognition, hypothesis generation, and problem-solving.
   2. Demonstrate the ability to systematically approach patient cases, considering relevant history, physical examination findings, and diagnostic data.
   3. Practice organizing and synthesizing clinical information to formulate a comprehensive patient assessment.
   4. Identify and apply key diagnostic principles, including recognizing red flags, considering differential diagnosis, and utilizing diagnostic tests effectively.
7. Electronic Medical Records (80 minutes)
   1. Navigate through various sections of the EMR system efficiently.
   2. Utilize EMR functionalities for tasks such as reviewing patient history, orders, results, and progress notes.
   3. Demonstrate an understanding of HIPAA regulations and the importance of patient confidentiality.
   4. Utilize search functions and filters to find specific clinical data efficiently.
   5. Engage in reflective practice to analyze the impact of accurate and thorough documentation on patient care.
8. Rounding/Presenting patients (90 minutes)
   1. Develop and deliver concise and structured patient presentations during rounds.
   2. Include pertinent information such as chief complaint, history, physical examination findings, assessment, and plan.
   3. Practice adjusting the level of detail in presentations for different clinical scenarios.
   4. Demonstrate efficient time management skills when presenting patients on rounds.
   5. Engage in simulated patient rounds to apply patient presentation techniques in a realistic setting.
9. Barriers to Care (115 minutes)
   1. Recognize the impact of building rapport on patient trust, satisfaction, and overall healthcare outcomes.
   2. Identify common communication barriers in healthcare, including language differences, cultural nuances, cognitive impairments, and sensory deficits.
   3. Develop awareness of personal biases and potential barriers to effective communication.
   4. Practice using body language, facial expressions, and gestures to convey empathy and understanding.
   5. Explore the use of technology, such as translation services and communication aids, to overcome language and hearing barriers.
   6. Emphasize the importance of involving patients in shared decision-making.
   7. Practice collaborative communication to empower patients to actively participate in their healthcare.
   8. Participate in simulated patient interactions to apply communication skills in realistic scenarios.
   9. Engage in reflective practice to analyze personal communication strengths and areas for improvement.
10. Tips & Tricks (50 minutes)
    1. Develop effective study strategies for clinical knowledge retention.
    2. Learn practical strategies for clinical efficiency and organization.
    3. Discuss approaches to common challenges encountered in clinical settings, such as time management and prioritization.
    4. Develop skills in navigating workflow and interprofessional collaboration.
    5. Explore strategies for effectively reviewing medical literature and staying updated on clinical guidelines.

References

1. Atherley A, Dolmans D, Hu W, Hegazi I, Alexander S, Teunissen PW. Beyond the struggles: a scoping review on the transition to undergraduate clinical training. Med Educ. 2019;53(6):559-570. doi:10.1111/medu.13883

2. Remmen R, Scherpbier A, Van Der Vleuten C, Denekens, J, Derese A, Herman I, et al. Effectiveness of basic clinical skills training programmes: A cross-sectional comparison of four medical schools. Med Educ. Published online 2001;35(2):121-128. doi:10.1046/j.1365-2923.2001.00835.x

3. Frye AW, Das Carlo M, Litwins SD, Karnath B, Stroup-Benham C, Lieberman SA. Effect of curriculum reform on students’ preparedness for clinical clerkships: a comparison of three curricular approaches in one school. Acad Med. 2002 Oct1;77(10 SUPPL.): S54-S57. doi:10.1097/00001888-200210001-00018

4. Van Hell EA, Kuks JBM, Borleffs JCC, Cohen-Schotanus J. Alternating skills training and clerkships to ease the transition from preclinical to clinical training. Med Teach. 2011;33(12):e689-96. doi:10.3109/0142159x.2011.611837

5. Jacobs JCG, Bolhuis S, Bulte JA, Laan R, Holdrinet RSG. Starting learning in medical practice: an evaluation of a new Introductory Clerkship. Med Teach. 2005 Aug;27(5):408-414. doi:10.1080/01421590500087001

6. Whipple ME, Barlow CB, Smith S, Goldstein EA. Early introduction of clinical skills improves medical student comfort at the start of third-year clerkships. Acad Med. 2006 Oct;81(10 Suppl):S40-43. doi:10.1097/00001888-200610001-00011

7. Norris TE, Schaad DC, DeWitt D, Ogur B, Hunt DD, Consortium of Longitudinal Integrated Clerkships. Longitudinal integrated clerkships for medical students: an innovation adopted by medical schools in Australia, Canada, South Africa, and the United States. Acad Med. 2009 Jul;84(7):902-907. doi:10.1097/acm.0b013e3181a85776

8. O’Brien BC, Poncelet AN. Transition to clerkship courses: preparing students to enter the workplace. Acad Med. 2010 Dec;85(12):1862-1869. doi:10.1097/acm.0b013e3181fa2353

9. Chumley H, Olney C, Usatine R, Dobbie A. A short transitional course can help medical students prepare for clinical learning. *Family Medicine*. 2005 Jul-Aug;37(7):496-501.

10. Stewart RA, Hauge LS, Stewart RD, Rosen RL, Charnot-Katsikas A, Prinz RA, Association for Surgical Education. A CRASH course in procedural skills improves medical students’ self-assessment of proficiency, confidence, and anxiety. Am J Surg. 2007 Jun;193(6):771-773. doi:10.1016/j.amjsurg.2007.01.019

11. Chittenden EH, Henry D, Saxena V, Loeser H, O'Sullivan PS. Transitional clerkship: an experiential course based on workplace learning theory. Acad Med. 2009 July:84(7):872-876. doi:10.1097/acm.0b013e3181a815e9

12. Sakai DH, Fong SFT, Shimamoto RT, Omori JSM, Tam LM. Medical school hotline: transition to clerkship week at the John A. Burns School of Medicine. Hawaii J Med Public Health. 2012 Mar;71(3):81-83.

13. Dehghani M, Athar O, Ashourioun V, Akhlagi MR, Avizhgan M, Esmaeili A, et al. A transitional curriculum for preparing medical students for internship, does it work? J Res Med Sci. 2013 Jun;18(6):506-509.

14. Blohm M, Krautter M, Lauter J, Huber, J, Weyrich P, Herzog W, et al. Voluntary undergraduate technical skills training course to prepare students for clerkship assignment: tutees’ and tutors’ perspectives. BMC Med Educ. 2014 Apr 4;14:71. doi:10.1186/1472-6920-14-71

15. Connor DM, Conlon PJ, O'Brien BC, Chou CL. Improving clerkship preparedness: a hospital medicine elective for pre-clerkship students. Med Educ Online. 2017;22(1):1307082. doi:10.1080/10872981.2017.1307082

16. Poncelet A, O’Brien B. Preparing medical students for clerkships: a descriptive analysis of transition courses. Acad Med. 2008 May;83(5):444-451. doi:10.1097/acm.0b013e31816be675

17. Knobloch AC, Ledford CJW, Wilkes S, Saperstein AK. The impact of near-peer teaching on medical students’ transition to clerkships. Fam Med. 2018 Jan;50(1):58-62. doi:10.22454/fammed.2018.745428

18. Nakazato T, Callahan Z, Kuchta K, Linn JG, Joehl RJ, Ujiki MB. A 1-day simulation-based boot camp for incoming general surgery residents improves confidence and technical skills. Surgery. 2019 Oct;166(4):572-579. doi:10.1016/j.surg.2019.05.051

19. Wayne DB, Cohen ER, Singer BD, Moazed F, Barsuk, JH Lyons EA, et al. Progress toward improving medical school graduates’ skills via a “boot camp” curriculum. Simul Healthc. 2014 Feb;9(1):33-39. doi:10.1097/sih.0000000000000001

20. Sonnadara RR, Garbedian S, Safir O, Mui C, Mironova P, Nousiainen, M, et al. Toronto orthopaedic boot camp III: examining the efficacy of student-regulated learning during an intensive, laboratory-based surgical skills course. Surgery. 2013 Jul;154(1):29-33. doi:10.1016/j.surg.2013.05.003

21. Gervais V, Grabs D, Bougie E, Salib GE, Bortoluzzi P, Tremblay DM. The Montreal Plastic Surgery Residency Bootcamp: structure and utility. Plast Reconstr Surg Glob Open. 2023 Oct;11(10):e5337. doi:10.1097/GOX.0000000000005337

22. Windish DM, Paulman PM, Goroll AH, Bass EB. Do clerkship directors think medical students are prepared for the clerkship years. Acad Med. 2004 Jan;79(1):56-61. doi:10.1097/00001888-200401000-00013

23. O’Brien B, Cooke M, Irby DM. Perceptions and attributions of third-year student struggles in clerkships: do students and clerkship directors agree? Acad Med. 2007 Oct;82(10):970-978. doi:10.1097/acm.0b013e31814a4fd5

24. Prince KJAH, Boshuizen HPA, Van der Vleuten CPM, Scherpbier AJJA. Students’ opinions about their preparation for clinical practice. Med Educ. 2005 Jul;39(7):704-712. doi:10.1111/j.1365-2929.2005.02207.x

25. Atherley AE, Hambleton IR, Unwin N, George C, Lashley PM, Taylor CG Jr. Exploring the transition of undergraduate medical students into a clinical clerkship using organizational socialization theory. Perspect Med Educ. 2016 Apr;5(2):78-87. doi:10.1007/s40037-015-0241-5

26. McKee A, Markless S. Using action learning sets to support students managing transition into the clinical learning environment in a UK medical school. Action Learn Res Pract. 2017;14(3):275-285. doi:10.1080/14767333.2017.1360933

27. Butts CA, Speer JJ, Brady JJ, Stephenson RJ, Langenau E, DiTomasso R, et al. Introduction to clerkship: bridging the gap between preclinical and clinical medical education. J Am Osteopath Assoc. 2019 Sep;119(9):578-587. doi:10.7556/jaoa.2019.101

28. Ryan MS, Feldman M, Bodamer C, Browning J, Brock E, Grossman C. Closing the gap between preclinical and clinical training: impact of a transition-to-clerkship course on medical students’ clerkship performance. Acad Med. 2020 Feb;95(2):221-225. doi:10.1097/acm.0000000000002934
